# Impact of AfterAMI Mobile App on Quality of Life, Depression, Stress and Anxiety in Patients with Coronary Artery Disease: Open Label, Randomized Trial

**DOI:** 10.3390/life13102015

**Published:** 2023-10-05

**Authors:** Maria Boszko, Bartosz Krzowski, Michał Peller, Paulina Hoffman, Natalia Żurawska, Kamila Skoczylas, Gabriela Osak, Łukasz Kołtowski, Marcin Grabowski, Grzegorz Opolski, Paweł Balsam

**Affiliations:** 1st Chair and Department of Cardiology, Medical University of Warsaw, 02-097 Warsaw, Poland; mariaboszko@gmail.com (M.B.);

**Keywords:** coronary artery disease, cardiac rehabilitation, myocardial infarction, quality of life, telemedicine, mobile health, smartphone, mobile application, mHealth, telehealth

## Abstract

mHealth solutions optimize cardiovascular risk factor control in coronary artery disease. The aim of this study was to investigate the influence of mobile app AfterAMI on quality of life in patients after myocardial infarction. 100 participants were randomized (1:1 ratio) into groups: (1) with a rehabilitation program and access to afterAMI or (2) standard rehabilitation alone (control group, CG). 3 questionnaires (MacNew, DASS21 and EQ-5D-5L) were used at baseline, 1 month and 6 months after discharge. Median age was 61 years; 35% of patients were female. At 1 month follow up patients using AfterAMI had higher general quality of life scores both in MacNew [5.78 vs. 5.5 in CG, *p* = 0.037] and EQ-5D-5L [80 vs. 70 in CG, *p* = 0.007]. At 6 months, according to MacNew, the app group had significantly higher scores in emotional [6.09 vs. 5.45 in CG, *p*= 0.017] and physical [6.2 vs. 6 in CG, *p* = 0.027] aspects. The general MacNew quality of life score was also higher in the AfterAMI group [6.11 vs. 5.7 in CG, *p* = 0.015], but differences in EQ-5D-5L were not significant. There were no differences between groups in the DASS21 questionnaire. mHealth interventions may improve quality of care in secondary prevention, however further studies are warranted.

## 1. Introduction

Ischemic heart disease is responsible for a considerable number of deaths in the global perspective [1]. Considering the growing prevalence of major cardiovascular risk factors (e.g., obesity, diabetes, hypertension, dyslipidemia) in modern society, the related economic health burden is expected to grow in the future. Even though optimal treatment of the acute phase of the disease is well managed, it is the long-term outcomes that are still unsatisfactory, with one-year mortality after an acute coronary event estimated at about 12% [2]. Coronary artery disease (CAD) may be associated with numerous unfavorable outcomes including chest pain, decreased exercise capacity, depression, rehospitalizations, recurrent myocardial infarction and stroke. As a result, suffering from a myocardial infarction often leads to poorer quality of life [3]. Therefore, exploring new effective strategies for secondary prevention is crucial.

Telemedicine provides physicians with several innovative solutions supporting cardiac rehabilitation in CAD patients. Standard therapeutical management can be augmented with telerehabilitation, wearable activity monitors, mobile apps, dedicated web pages, online dietary consultations, video psychological coaching and many more. According to European Society of Cardiology (ESC) guidelines, telehealth and mHealth interventions may be considered to improve patient participation in cardiac rehabilitation (CR) and long-term adherence to healthy habits (Class IIb) [4].

The benefits of adopting these novel treatment strategies have been thoroughly documented. For instance, in remote regions telerehabilitation enabled overcoming geographical barriers and increased attendance [5]. What is more, these interventions have been proven to be successful in promoting health literacy among patients, resulting in better lipid and glycemic control, smoking cessation, as well as improving physical activity habits [6,7,8]. Optimizing cardiovascular risk factors control using telemedicine has led to a reduction in rehospitalizations and adverse events, as a cost-effective alternative to standard models of care [9,10]. Interestingly, decreased health-related quality of life after myocardial infarction has also been associated with a risk of hospital admissions, major cardiovascular events, and death [11]. However, data regarding the impact of mobile apps on CAD patients’ quality of life remains scare.

## 2. Materials and Methods

**Figure 1:**
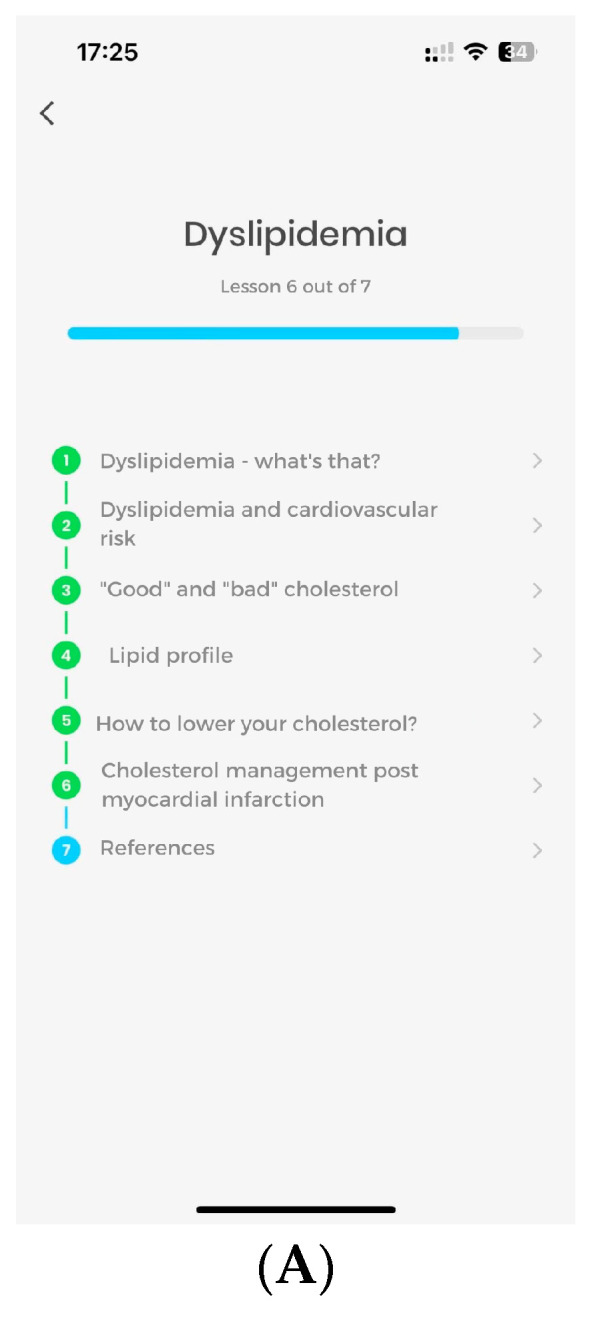

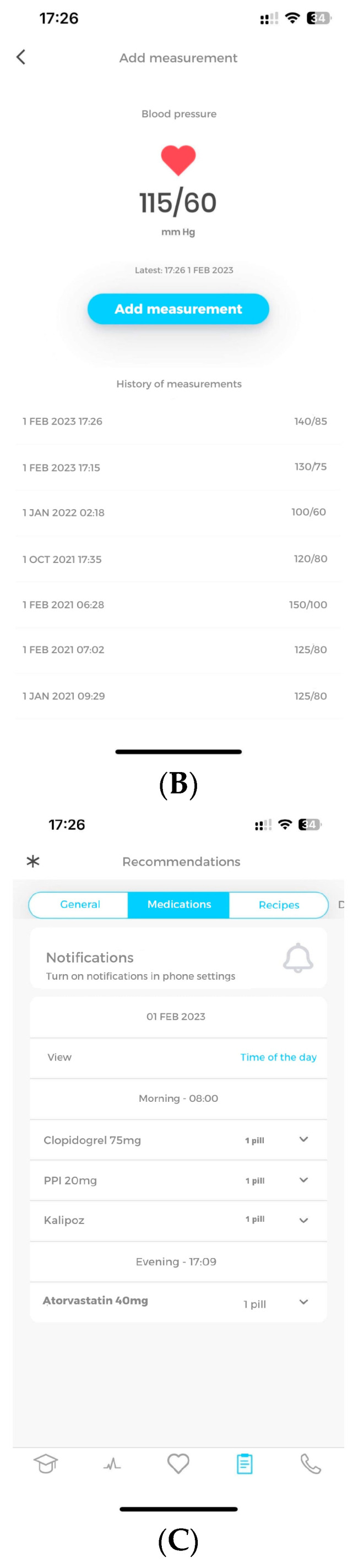
(**A**–**C**). Example screenshots of the afterAMI app.

The data used in this study comes from a randomized, open afterAMI trial (Mobile App and Digital System for Patients After Myocardial Infarction)-number NCT04793425 in ClinicalTrials.gov. The protocol for this study was previously published [12]. The Institutional Review Board approval has been granted (KB/150/2020). The inclusion criteria comprised of: age ≥ 18 years, written informed consent, possession of smartphone (iOS/Android), hospital admission owing to acute myocardial infarction, basic ability to use mobile apps (positive skill test, designed by the authors). The exclusion criteria were: life expectancy < 6 months due to a non-cardiac disease, pregnancy or breastfeeding, negative test results (everyday mobile application use), age < 18 years old, lack of signed informed consent, lack of a mobile phone with Internet access and Android/iOS. The study was conducted in the 1st Chair and Department of Cardiology, Medical University of Warsaw. 100 consecutive, eligible patients presenting with acute myocardial infarction were recruited. The patients were then randomized into (1) a control group (CG) receiving optimal standard of care or (2) an interventional group (IG) provided with telemedical support in the form of a mobile application (afterAMI), using 1:1 ratio. The app consisted of numerous features including vital signs panel, educational materials on cardiovascular risk factors, drug taking reminders and patient-physician chat (Figure 1). The patients were followed up at 1-month and 6-months post discharge. A detailed description of the study design has been thoroughly described [12].

Quality of life was measured using 2 validated questionnaires: MacNew and EQ-5D-5L. Dass21 questionnaire was used to assess the severity of depressive symptoms. The following were collected at 3 timestamps: at baseline, at 1 month and 6 months after discharge. Each time the patients individually completed the printed paper versions of the questionnaires. The outbreak of the COVID-19 pandemic occurred within the duration of our research. As a result some patients decided to withdraw from the study, considering the risk of infection related with follow up visits. Still, the use of remote telephone communication enabled collection of crucial data.

MacNew includes 27 questions regarding physical, emotional, and social aspects of patient’s quality of life. The maximum score in every domain is 7 (high quality), and the minimum is 1 (poor quality) [13]. It has been validated in the Polish population [14].

The EQ-5D-5L questionnaire concerns five domains: mobility, self-care, everyday activity, pain and/or discomfort, and anxiety and/or depression [15]. Each aspect is assessed on a 5-degree scale: 1—indicating no problems within the aspect; 2—indicating slight problems; 3—indicating moderate problems; 4—indicating severe problems; 5—indicating an inability to e.g., move or extreme problems with the questioned aspect. At the end of the questionnaire the patients are presented with a visual analogue scale (VAS), where they are asked to indicate a score from 0 to 100, best describing their current health status (0 being the worst imaginable health state and 100 being the best). The mean VAS scores across different phases were also computed to see if there was a perceived change in the quality of life of patients before or after using the app.

In order to assess depression, anxiety and stress the patients were asked to complete the DASS 21 questionnaire [16]. It provides a quantitative measure of distress, as a sum of seven statements regarding each: depression, anxiety, and stress. Every statement has 4 possible answers with the following scoring: 0—did not apply to me at all/never, 1—applied to me to some degree, or some of the time/sometimes, 2—applied to me to a considerable degree/or a considerable time/often, 3—applied to me very much/or most of the time/almost always. The higher result in each section the greater the severity of depression, anxiety and stress, respectively.

Statistical analysis was performed by a blinded investigator using SAS. Testing for normalcy was performed using the Shapiro-Wilk test. Continuous variables with normal distribution were presented as mean and standard deviation (SD), non-normal distribution variables were reported as median and interquartile range (IQR). Comparison of variables with normal and non-normal distributions was performed using Student’s t-test and the non-parametric Mann–Whitney U test. Qualitative variables were compered using Fisher’s exact test. In addition, we assessed the change from baseline in case of quantitative variables. Upon completion of the final follow-up visits, we applied a per-protocol analysis to our calculations. All recruited patients (irrespective of study completion) were included for baseline population analysis. If the collected data was not complete, the patients were excluded from the particular analysis.

## 3. Results

Baseline patient characteristics are presented in Table 1. The patients’ median age was 61 years and 65% of the subjects were male. One patient in the control group did not receive the assigned intervention due to death during the initial hospitalization. In addition, at 1 month after discharge the number of patients lost to follow up was 11 (5 in IG and 6 in CG), accounting for a 11% attrition rate. 14 individuals were lost to follow-up at 6 months after discharge (8 in IG and 6 in CG). A total of 74 patients (37 in IG, 37 in CG) have completed the final follow up of the study. The studied groups did not display significant differences at baseline with regard to sociodemographic characteristics (sex, body weight, and general cardiovascular risk factors, cardiac rehabilitation). The individuals assigned to the CG were older (63.42 years old vs. 56.8) than those in the intervention group (*p* = 0.002). Heart failure and atrial fibrillation were more prevalent in the CG. In a previous study, we have reported lower LDL cholesterol levels in the “afterAMI” group (*p* < 0.001) at 1 month follow up, despite no differences at baseline [17]. Furthermore, at 6 months participants with digital support had fewer rehospitalizations and/or unplanned ambulatory visits, but our results did not achieve statistical significance (3 [8%] in AfterAMI vs. 10 [27%] in control group, *p* = 0.064) [18].

**Figure 2:**
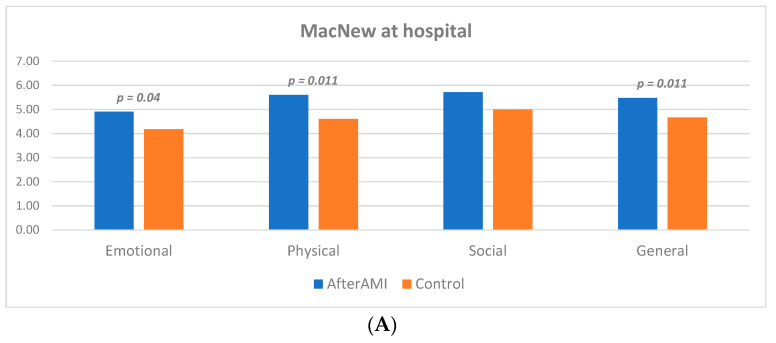

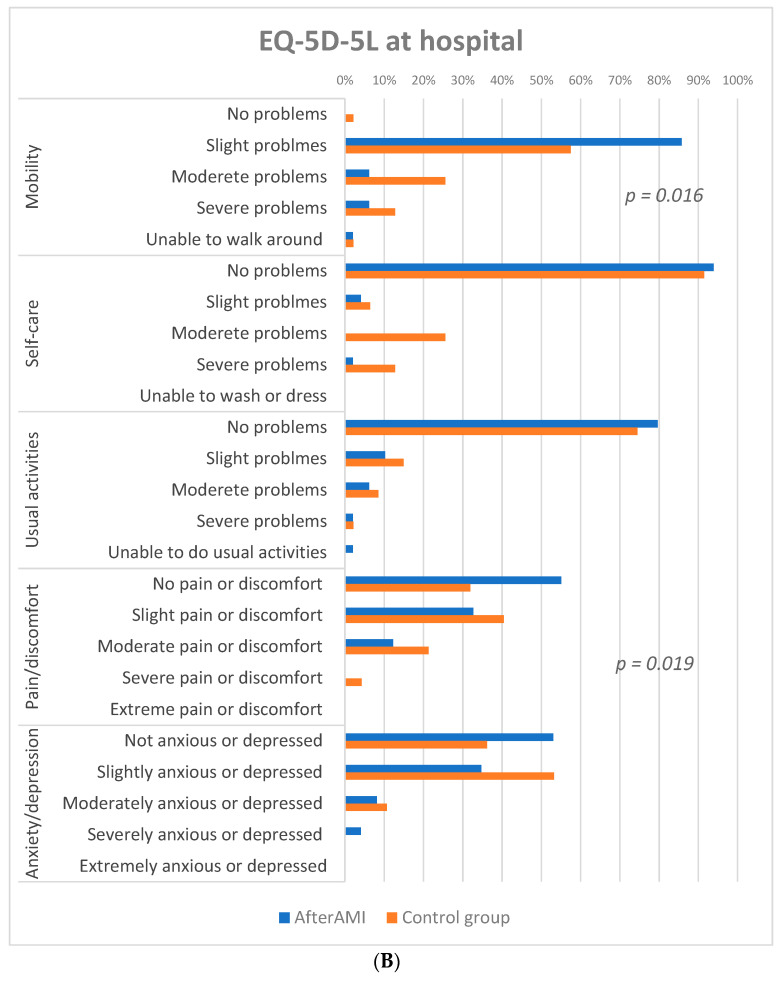
Baseline patient characteristics with regard to quality of life (**A**) MacNew questionnaire; (**B**) EQ-5D-5L questionnaire.

Regarding quality of life, some significant differences were observed at baseline in favor of the AfterAMI group (Figure 2). According to the MacNew questionnaire, patients in the intervention group demonstrated higher scores in questions regarding emotional [4.90 (4.09–5.63) vs. 4.18 (3.63–5.18), *p* = 0.040] and physical [5.6 (4.6–6.6) vs. 4.6 (3.8–5.4) *p*= 0.011] aspects, as well as in the general score [5.48 (4.81–5.92) vs. 4.67 (4.07–5.46) *p* = 0.011] (Figure 2A). Similarly, according to the EQ-5D-5L questionnaire more patients in the group provided with digital support reported lack of pain (*p* = 0.019) (Figure 2B). The reported VAS scale was also higher in this group [80 (60–90) vs. 69 (50–80) in CG, *p* = 0.015].

**Figure 3:**
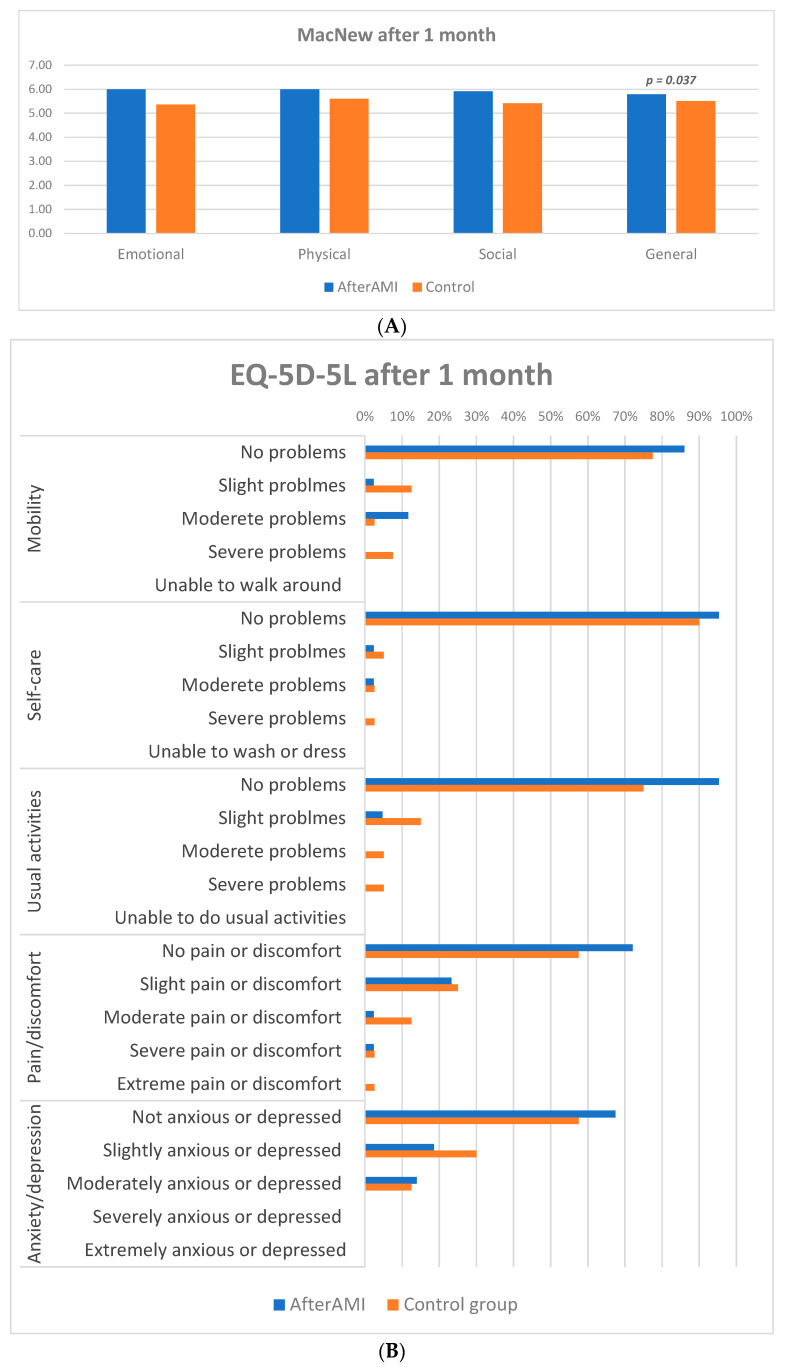
Quality of life assessment at 1 month follow up. (**A**) MacNew questionnaire; (**B**) EQ-5D-5L questionnaire.

Figure 3 presents results of quality of life assessment at 1 month follow up. After 30 days post discharge fewer significant differences were observed. According to MacNew questionnaire, patients receiving digital support still reported significantly higher general scores of quality of life [5.78 (5.37–6.3) vs. 5.5 (4.81–6.05) in CG, *p* = 0.037] (Figure 3A). However, at this time point other individual scores in each domain (emotional, physical and social aspects) were similar in both groups. Likewise, the only significant difference in the EQ-5D-5L questionnaire was the higher general score in the intervention group [80 (70–90) vs. 70 (60–80) in CG, *p* = 0.007], which was similar to baseline values (Figure 3B).

**Figure 4:**
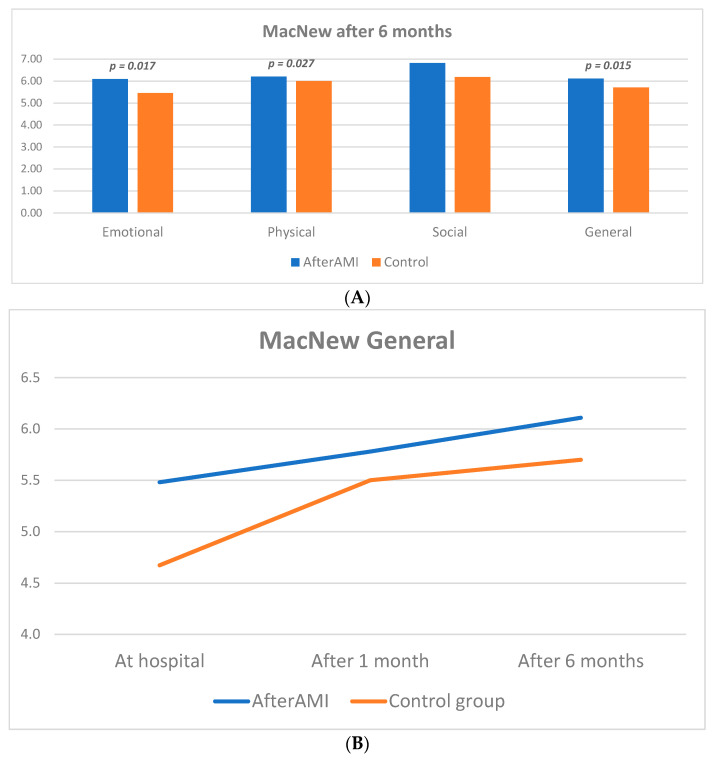

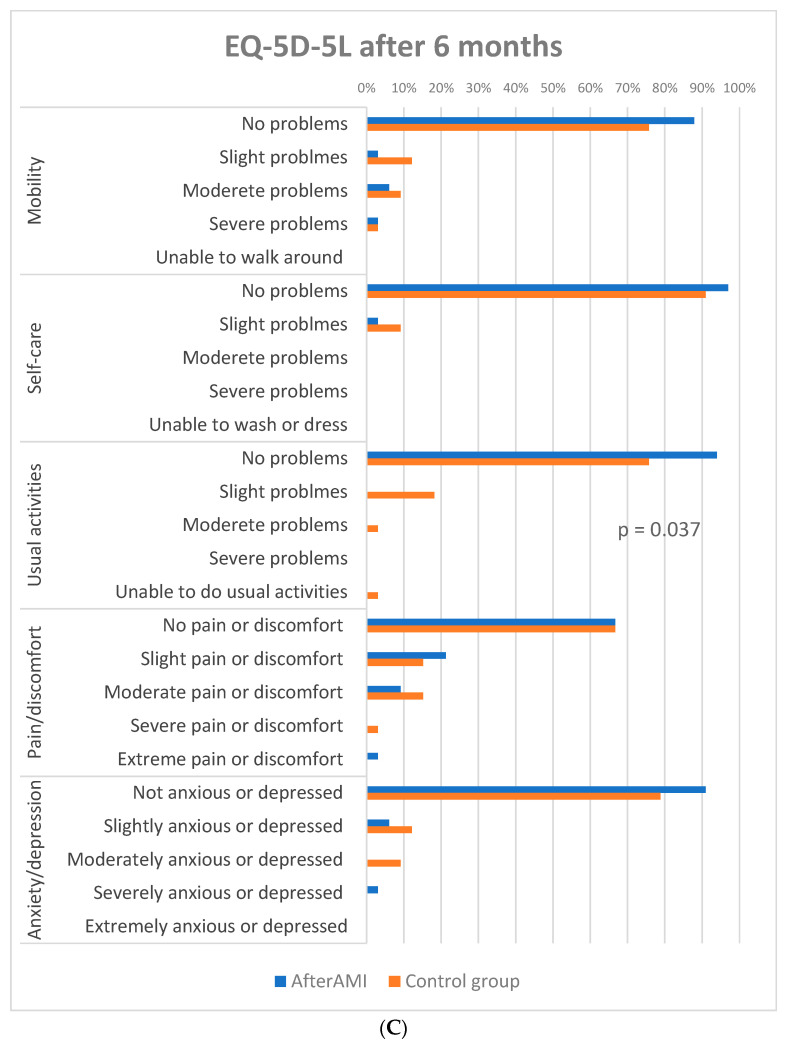
Quality of life assessment at 6 month follow up (**A**) MacNew questionnaire; (**B**) Dynamics of change in the general score of MacNew questionnaire; (**C**) EQ-5D-5L questionnaire.

In the final assessment at 6 months post discharge according to the MacNew questionnaire the app group had significantly better quality of life as expressed in emotional [6.09 (5.45–6.45) vs. 5.45 (4.73–6) in CG, *p* = 0.017] and physical [6.2 (5.8–7) vs. 6 (5.2–6.4) in CG, *p* = 0.027], but not social aspects (Figure 4A). Overall, the general MacNew quality of life score was also higher in the intervention group [6.11 (5.63–6.55) vs. 5.7 (5.26–6) in CG, *p* = 0.015], which was in line with the trend demonstrated both at baseline and at 1-month follow-up (Figure 4B). On the basis of the EQ-5D-5L questionnaire, more patients in the control group had some problems with performing usual activities (*p* = 0.037) (Figure 4C). There were no significant differences between groups on the basis of the DASS21 questionnaire, neither at baseline, nor at any follow-up.

## 4. Discussion

The results of our study suggest that digitally supported traditional cardiac rehabilitation translates into better quality of life in patients at 6 months after myocardial infarction according to MacNew questionnaire, but not EQ-5D-5L. Furthermore, we report that throughout the duration of the study the groups displayed similar levels of anxiety and depression.

Early anxiety and depression rates after a cardiac event are estimated at as high as 43% and 22%, respectively [19]. These however may continue for several months after, leading to poorer health outcomes, including higher morbidity and mortality [20,21]. Secondary prevention is key for long-term care in CAD patients, including improving quality of life [22]. It is worth noting, that using e-Health solutions (including mobile apps) to improve psychosocial health has been recommended by the European Society of Cardiology (ESC) as a form of secondary prevention in CAD patients [23,24].

Even though quality of life is an important outcome, it is often not captured in most clinical trials, which has been stressed in recent guidelines [25]. There are some studies describing the influence of mHealth on quality of life in CAD patients. Recent meta-analysis involving 20 studies and a total sample size of 4535 patients showed that both the physical and mental dimensions of quality of life were better in the mHealth group, but the differences in the general scores did not reach statistical significance (*p* = 0.15) [26]. However, quality of life was studied in only 39% (9/23) of these randomized controlled, trials.

The SUPPORT study investigated the use of smartphone app on cardiovascular lifestyle and mediation adherence in 174 myocardial infarction patients. The change in lifestyle and quality of life measured with European Quality of Life–5 Dimensions after a 6 months period was not significant [27]. Even though the mobile app and the investigated population were similar to ours, the control group in this study was provided with a simplified tool containing a drug adherence e-diary, which was not the case in our research. In another paper, a patient-tailored motivational HeartHab app contributed to a perceived improvement in quality of life demonstrated by a mean increase of 0.06 in quality-adjusted life years on the basis of the EQ-5D and a mean decrease of 7.14% in anxiety during the app usage phase [28]. However, the power of this mixed-methods study may be limited by a small sample size, consisting of only 32 participants and a short observation period of only 4 months. Our analysis showed no differences between groups with regard to anxiety levels and it did not compere the quality-adjusted life years between groups. However, higher general scores according to EQ-5D-5L in the digitally supported group were demonstrated. Yet our research involved a larger sample size and longer observation period. Furthermore, it did not apply a cross-over approach and did not investigate the influence of the app on patient motivation for sustaining a healthy lifestyle. Another study involving patients using smartphone-guided training system reported comparable anxiety and depression rates (HADS questionnaire) and quality of life (EQ-5D) between the studied groups [29]. Even though our results also demonstrate no significant differences regarding anxiety measured by a different questionnaire (DASS21), a trend towards higher scores in depression, anxiety and stress in the control group was observed. What is more, we demonstrated differences in the EQ-5D score. However, the mobile app design in this study was dedicated to guiding exercise training and not improving cardiovascular knowledge like the AfterAMI app that we used. In another trial, a group of researchers in Norway used the Vett^®^ application in a study involving 113 participants [8]. After 1-year follow-up the groups displayed similar scores in EQ VAS and HeartQol questionnaire. However, there was a significant change from baseline in the intervention group, but only with regard to EQ VAS and the emotional aspect of HeartQol. Compering these results with ours, one should consider that the study population the authors investigated was less homogenic than ours and included patients after valve surgery, as well as other heart diseases. Moreover, our observation period did not exceed 6 months. Finally, the Vett^®^ app was more interactive and engaging, with features like personalized goal setting, feedback reports and weekly ratings of goal achievements, which was not the case with AfterAMI. These findings show that the effects of digitally augmented secondary prevention on quality of life in CAD patients tend to vary. What is more, often the individual components of the EQ questionnaire are not presented in the articles, most likely due to lack of significant differences between groups, which is in line with our findings. However, in our study we demonstrated a trend towards a significantly higher general EQ VAS in the AfterAMI group throughout the duration of the study.

It is worth stressing, that most studies assess quality of life after a period of observation or show no differences between groups at baseline. However, in our research we showed, that digitally supported patients have higher quality of life yet before discharge. This may be a result of a subjective feeling of receiving better care, as the patients are provided with additional monitoring tools. Perhaps the vision of receiving extra support in following recommendations and/or the possibility of more frequent interactions with the physician have provided the patients with enough comfort to alleviate the acute distress. Conducting a blinded study would provide more information on this matter, however it is not feasible when using a mobile app as the investigated intervention. Further studies should focus on researching this phenomenon.

Experiencing a severe life-threatening event like myocardial infarction may lead to depression. Early diagnosis is crucial, as presence of depressive symptoms post MI is associated with an increased risk of mortality [30]. Another concern are suboptimal treatment results in the CAD population [31]. The two diseases may also be connected at the pathophysiological level. Pizzi et al. demonstrated improved endothelial function and reduced inflammatory markers due to sertraline treatment in patients with coronary heart disease [32]. Implementing mHealth solutions could play an important role in improving care in these patients in the future. We presented no differences between groups on the basis of the DASS21 questionnaire, neither at baseline, nor at any follow-up. This finding may be due to the small sample size or the subjective nature of the questionnaire and its limitations. However, other possible explanations should be explored in following trials.

Finally, quality of live has been demonstrated to have predictive value for rehospitalization, as well as all-cause mortality [33,34]. Therefore, addressing this aspect in further mHealth studies is warranted.

There are some study limitations that should be noted. Firstly, there was a substantial attrition of participants over time, from 100 at baseline to 26% loss by the final follow-up. In the afterAMI group heart failure was less prevalent and the patients were younger. In addition, in the afterAMI cohort we observed higher prevalence of KOS-rehabilitation program and more patients in this group were male and employed. Even though these factors could have some impact on the outcome, the differences between groups were not statistically significant. Furthermore, it was not feasible to conduct a blinding process, due to the nature of this study. Lastly, the outbreak of the COVID-19 pandemic occurred within the duration of our research. As a result some patients decided to withdraw from the study, considering the risk of infection related with follow up visits. Still, the use of remote telephone communication enabled collection of crucial data.

## 5. Conclusions

Augmenting the traditional model of cardiac rehabilitation with mobile AfterAMI app has led to improved quality of life in CAD patients, according to the MacNew questionnaire. mHealth interventions hold promise to further improve the quality of care in secondary prevention, however further studies are warranted.

## Figures and Tables

**Table 1:** Patient characteristics.

Variable		AfterAMI	Control	*p*-Value
Clinical data				
Age [years]		56.8 ± 9.23	63.42 ± 11.4	**0.0019**
BMI [kg/m^2^]		28.5 ± 4.06	28.11 ± 5.38	0.7247
Body weight		88.95 ± 13.86	85.47 ± 24.33	0.4625
Sex		34 (68%)	31 (61%)	0.6753
KOS—rehabilitation		17 (34%)	9 (18%)	0.1095
Hospitalization [days]		6 (4–8)	7 (5–11)	0.2143
STEMI		25 (50%)	20 (40%)	0.4176
NSTEMI		25 (50%)	30 (60%)	0.4176
Infarction artery	LAD	26 (52%)	24 (48%)	1
	LCA	15 (30%)	17 (34%)	0.5218
	RCA	16 (32%)	24 (48%)	0.1438
PTCA		39 (78%)	39 (78%)	0.6222
Bypass surgery		5 (10%)	6 (12%)	0.7589
Body weight		88.9459 ± 13.8663	85.4657 ± 24.3327	0.4625
Height [cm]		176.3 ± 7.2328	171.6 ± 8.9729	**0.0186**
Nicotinism		33 (66%)	32 (64%)	1
Packet years		20 (0–30)	14 (0–32.5)	0.7934
Diabetes, type I		2 (4%)	0 (0%)	0.4949
Diabetes, type II		11 (22%)	11 (22%)	1
Hypertension		30 (60%)	34 (68%)	0.3828
Dyslipidemia		36 (72%)	39 (78%)	0.3069
Atrial fibrillation/atrial flutter		1 (2%)	7 (14%)	**0.0288**
Heart failure		6 (12%)	15 (30%)	**0.0274**
Implanted pacemaker or ICD		1 (2%)	5 (10%)	0.1112
Chronic kidney disease		1 (2%)	1 (2%)	1
Peripheral artery disease		1 (2%)	1 (2%)	1
EF in hospital [%]		51.78 ± 8.42	48.0 ± 9.22	**0.0394**
CVD risk factors knowledge		8 (6–9)	8 (4–9)	0.4131
Employed		27 (54%)	17 (34%)	0.1261
Lab tests at hospital				
Troponin I [μg/L]		0.7930 (0.2250–5.5710)	0.694 (0.111–4.350)	0.7248
Troponin II [μg/L]		2.2550 (0.7145–8.7340)	5.640 (0.437–34.635)	0.1702
Creatinine [mg/dL]		0.98 ± 0.21	1.05 ± 0.34	0.1991
eGFR [mL/(min × 1.72 m^2^)]		79.16 ± 17.22	73.28 ± 20.93	0.1351
Na [mmol/L]		139.1 ± 3.05	139.6 ± 4.36	0.5399
K [mmol/L]		4.17 ± 0.45	4.38 ± 0.51	**0.0363**
WBC [×10^9^/L]		10.27 ± 3.04	10.19 ± 2.94	0.9052
HbA1C [%]		5.8 (5.4–7.1)	5.6 (5.4–6.0)	0.4593
NTproBNP [pg/mL]		422 (133–1256)	886.5 (230–2250)	0.0735
HgB [g/dL]		14.58 ± 1.49	14.14 ± 1.83	0.1989
Total cholesterol [mg/dL]		191.3 ± 71. 57	192.1 ± 52.29	0.9523
HDL [mg/dL]		39.55 ± 10.02	46.78 ± 10.65	0.0010
LDL [mg/dL]		117.5 ± 68.59	111.7 ± 61.56	0.6621
Tg [mg/dL]		146 (92–233)	136.5 (87–201)	0.2423
Drugs at discharge				
ACEi		42 (84%)	40 (80%)	0.5229
ARB		4 (8%)	2 (4%)	0.2314
ARNI		0 (0%)	0 (0%)	
MRA		9 (18%)	15 (30%)	0.2366
B-blocker		42 (84%)	41 (82%)	0.7398
CCB		20 (40%)	10 (20%)	**0.0257**
Statin		46 (92%)	45 (90%)	1
Ezetimibe		5 (10%)	2 (4%)	0.2673
VKA		0 (0%)	0 (0%)	
NOAC		1 (2%)	2 (4%)	1
ASA		45 (90%)	43 (86%)	1
Clopidogrel		12 (24%)	13 (26%)	1
Prasugrel		2 (4%)	0 (0%)	0.2419
Ticagrelor		28 (56%)	28 (56%)	1
Digoxin		0 (0%)	0 (0%)	

*p* valuves < 0.05 are in bold. ACEi—angiotensin-converting-enzyme inhibitors, ARB—angiotensin receptor blockers, ARNI—angiontensin receptor neprilysin inhibitor, ASA—acetylsalicylic acid, BMI—body mass index, CVD—cardiovascular disease, EF—ejection fraction, eGFR—estimated glomerular filtration rate, HbA1C—hemoglobin A1c, HDL—high-density lipoprotein, ICD—implantable cardioverter-defibrillator, KOS—Rehabilitation—Coordinated Care Program for patients after myocardial infarction; LAD—left anterior descending artery, LCA—left circumflex artery, LDL—low-density lipoprotein, MRA—aldosterone receptor antagonists, CCB—calcium channel blockers, NOAC—novel oral anticoagulants, NSTEMI—Non-ST–elevation myocardial infarction, NTproBNP—N-terminal pro–B-type natriuretic peptide, PTCA—percutaneous transluminal coronary angioplasty, RCA—right coronary artery, STEMI—ST-elevation myocardial infarction, Tg—triglycerides, VKA—vitamin K antagonist, WBC—white blood cells.

## Data Availability

The data underlying this article will be shared on reasonable request to the corresponding author.
